# COVID-19 pandemic reveals the peril of ignoring metadata standards

**DOI:** 10.1038/s41597-020-0524-5

**Published:** 2020-06-19

**Authors:** Lynn M. Schriml, Maria Chuvochina, Neil Davies, Emiley A. Eloe-Fadrosh, Robert D. Finn, Philip Hugenholtz, Christopher I. Hunter, Bonnie L. Hurwitz, Nikos C. Kyrpides, Folker Meyer, Ilene Karsch Mizrachi, Susanna-Assunta Sansone, Granger Sutton, Scott Tighe, Ramona Walls

**Affiliations:** 1 University of Maryland School of Medicine, Institute for Genome Sciences, Baltimore, MD USA; 2 Australian Centre for Ecogenomics, The University of Queensland, Brisbane, Queensland Australia; 3 Gump South Pacific Research Station, University of California Berkeley, Moorea, French Polynesia; 4 Department of Energy, Joint Genome Institute, Berkeley, California 94598 USA; 5 European Bioinformatics Institute (EMBL-EBI), Wellcome Genome Campus, Hinxton, UK; 6 GigaScience, BGI-Hong Kong, NT, Hong Kong; 7 University of Arizona, Tucson, AZ USA; 8 Argonne National Laboratory, Argonne, Illinois USA; 9 National Center for Biotechnology Information, National Library of Medicine, National Institutes of Health, Bethesda, MD 20894 USA; 10 Oxford e-Research Centre, Department of Engineering Science, University of Oxford, Oxford, UK; 11 J. Craig Venter Institute, Rockville, Maryland USA; 12 University of Vermont, Burlington, Vermont USA

**Keywords:** Research data, Research management, Standards, Data integration

## Abstract

Efficient response to the pandemic through the mobilization of the larger scientific community is challenged by the limited reusability of the available primary genomic data. Here, the Genomic Standards Consortium board highlights the essential need for contextual genomic data FAIRness, for empowering key data-driven biological questions.

A research program at the University of Oxford, “Our World in Data”, maintains a global database on testing for COVID-19. Asked whether there are ‘low-hanging fruit’ to improve the response to the pandemic, Program Director Max Roser had a very simple answer: “*for all those who publish original data, provide a clear description of your data” (@MaxCRoser:* 1:39am · 12 Apr 2020 · Twitter Web App), highlighting the importance of maximizing the reusability of data. In the age of COVID-19, we are seeing where value really lies. Describing the WHO, WHAT, HOW, WHERE, and WHEN of genomic data enables comparative analysis, informs public health responses, drives assessment of outbreak progression and reveals variation in the host-specificity, modes of transmission, and sample collection protocols.

The cost of insufficiently describing information about the human host and collection process from genomic studies is greater than just the missing fields in a biological sample or nucleotide sequence record. Loss of critical genomics data reduces the near and long term utility of the data and hampers clinical advancements in risk prediction, diagnosis, treatment options and outcomes.

Descriptions of data are known as metadata. It is an unglamorous corner of science, but metadata standards are vital infrastructure – often holding the key for data-driven research discoveries. Yet, like much critical infrastructure, standards are little appreciated until crisis hits. The Genomic Standards Consortium (GSC, www.gensc.org) was founded 15 years ago by scientists observing that genome sequence data, still somewhat of a novelty at the time, rarely had the most basic metadata readily available in a structured format^1^. As the field evolved from primarily laboratory-based (highly controlled) biomedical studies towards studies of the natural world, variability in the environmental context of the study – notably around sample collection – became increasingly pertinent to the interpretation of results in addition to metadata on other aspects, such as laboratory methods. As a new breed of “molecular ecologists” studying natural systems arose, the availability of such temporal-spatial metadata became crucial for the interpretation of sequence data. For metagenomics studies (profiling all genetic material, usually microbial, in a given environment), the need for metadata was most obvious, as without it, the sequence data were largely uninterpretable. Our growing appreciation of the complex interactions between genes and environment (and where appropriate host) in determining phenotypes compels a greater understanding of the environmental context of any sequence.

Which metadata were needed to address key biological questions across genomic studies was unknown and undefined at the time. Should researchers provide everything possible or at least a minimal set of information that was applicable to all types of current and future studies? If the latter, what is the reasonable minimum and who would set that standard? The GSC was formed to address this question^2^. The first checklists devised by the GSC focused on guiding scientists to add the minimal information required to enable re-use of their data in future studies^3^. The standards were subsequently expanded into the suite of MIxS (Minimum Information about any (x) Sequence) checklists to provide minimal and expanded sets of metadata terms across different environment types for metagenome and genome studies^4^. MIxS checklists are also recommended by a number of journals, and implemented by a growing set of international databases, as tracked in the MIxS record in FAIRsharing (https://fairsharing.org/FAIRsharing.9aa0zp).

Since the publication of the FAIR Principles^5^, which emphasize the importance of enhancing the ability of machines to automatically discover and use data and metadata, data management has been catapulted onto the international stage as a key component of open science^6^. Community standards for citing, reporting and sharing data, software, code, models, and other digital objects are taking centre stage in many global initiatives and domain specific alliances (e.g. Research Data Alliance, https://www.rd-alliance.org/groups/rda-covid19; Global Alliance for Genomics and Health, https://www.ga4gh.org; MetaSUB^7^: https://pangea.gimmebio.com/contrib/metasub)^8^. Few standards, however, related to data sharing and management practices exist. FAIRsharing^9^ provides an informative and educational snapshot of the standards landscape, tracking their life-cycle status and usage in databases and repositories, and their adoption by journals and funders’ data policies. Although the scientific community, funding agencies, and scholarly publishers endorse the concept that community-defined data and metadata standards underpin data reproducibility and enable FAIR data, putting them in action and complying with them takes time and effort by both individual researchers and community-based standards organizations.

To be FAIR, data must be published in a trustworthy repository. Despite widespread requirements to submit sequence data to a repository before publication, identifying sequence data for reuse is still severely limited by the lack of metadata submitted to genomic data repositories. For example, in the International Nucleotide Sequence Database Collaboration (INSDC, www.insdc.org) there are 2.1 million Sequence Read Archive (SRA) experiments listed under the taxonomy term “metagenomes”, less than 33% of which are tagged with environment metadata. Although published descriptions of metagenomic datasets are generally associated with enriched metadata describing the environment, source material, and sequencing technology, and in theory it is possible for one to read the manuscripts (including figures, tables and supplementary information) and gather that information, this is an onerous task when dealing with multiple studies. It also means multiple researchers potentially repeating the same work of trawling for metadata, resulting in significant researcher-hours that could be better spent actually interrogating the data.

With COVID-19, the time and place a biosample was collected has suddenly become a life and death issue. As with previous pathogen outbreaks, the reporting of pertinent metadata has become critical. The time and effort to describe data requires researchers to value the effort for the Greater Good (and for society to reward their effort), to have knowledge on selecting the appropriate metadata types, to integrate metadata standardization in data management plans and research workflows, to prioritize community-driven efforts towards defining and implementing metadata standards, and the development of enhanced informative user guidelines. Despite the implementation of the breadth of (N = 20) MIxS packages (and their associated minimal contextual information requirements) across the INSDC partners (NCBI, EMBL-EBI, DDBJ)^10^ and core bioinformatics pipelines/web applications (e.g. GenBank, European Nucleotide Archive (ENA), DNA Data Bank of Japan (DDBJ), National Genomics Data Center, European Genome-phenome Archive (EGA), QIIME, Genomes OnLine Database (GOLD), MGnify, MG-RAST)^11–15^, poorly described data are still all too common across genomic and metagenomic studies. This is exemplified when data submitters provide only partial or mismatched metadata by leaving fields blank or filling in ‘missing’ (Fig. 1) for nucleotide records (in NCBI’s GenBank (https://www.ncbi.nlm.nih.gov/nucleotide/) or EMBL-EBI’s European Nucleotide Archive (ENA)) or biological sample records (in NCBI’s BioSample https://www.ncbi.nlm.nih.gov/biosample/ or EMBL-EBI’s BioSamples https://www.ebi.ac.uk/biosamples/). For example, “host” is not annotated in 2,416 of the 5,198 SARS-CoV-2 BioSample submissions.

**Fig. 1:**
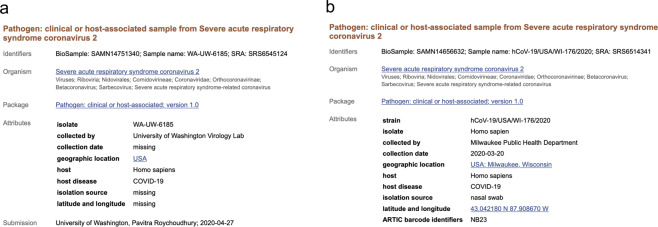
Lost opportunities for data reuse, SARS-CoV-2 (txid2697049[Organism:noexp]) BioSample records, where (**a**) **collection date** = “missing”: 143; **latitude and longitude** = “missing”: 1375; (**b**) SARS-CoV-2 BioSample record with complete metadata.

Responsible sharing of genomic and health-related data must, of course, recognize that genomic data are highly sensitive and identifiable. Reasonable steps must be taken to remove or obscure key information that may make sample data traceable to an individual person, such as only reporting the year collected and reporting geographic subdivisions no more specifically than a first-level administrative division (e.g. state)^16^.

Even when researchers use the required metadata packages in INSDC, reporting of critical metadata is often hampered by confusion over the selection of metadata packages and inconsistent value specification for specific metadata terms, leading to the submission of incomplete, mislabeled, or missing metadata. As exemplified by 5,198 SARS-CoV-2 BioSample submissions (as of May 4, 2020), samples are being submitted using primarily the Pathogen: clinical or host-associated package, with a small set of submissions using the Microbe, Virus, or human-associated MIxS packages. The requirements for specific metadata attributes should ensure that sufficient contextual information is included. However, submitters may provide inappropriate information in these fields at the time of submission.

In an example relevant to COVID-19, the more granular level taxon “viral metagenome” in the INSDC SRA has about 12k experiments (12,105 runs)(as of 5/7/2020). Of those (viewed in SRA Run Selector: https://www.ncbi.nlm.nih.gov/Traces/study/), 68% (8,225/12,105) have no reported geo_loc_name (country/continent) and 9% of runs have an ‘uncalculated’ geo_loc_name, as the submitting institution information has been filled in the country/continent field. Perhaps encouragingly, SARS-CoV-2 (txid2697049) in the SRA identifies 3,352 records with (SRA Run Selector) only 25% (887) of the 3,352 runs are reported with no country/continent metadata and only one submission with an ‘uncalculated’ geo_loc_name. Regrettably, we simply do not know the geographic origin of many sequenced samples, which is critical for subsequent analysis and data reuse.

The majority of samples annotating the ‘disease’ metadata field include the World Health Organization (WHO) nomenclature “COVID-19”. However, the variation in submissions for ‘host disease’ complicate further analysis, as human disease has been submitted as (number of samples): COVID-19 (2,243); severe acute respiratory syndrome (119); Acute infection (34); novel coronavirus pneumonia (11); nCoV pneumonia (8); COVID19 (6); pneumonia (5); respiratory infection (2); Covid-2019 (2); Severe acute respiratory syndrome coronavirus 2 (1); pneumonia complicated by diarrhea (1). More than half of the submitted samples do not report any disease (2,766). Standard annotation of the metadata is supported by the usage of the structured controlled vocabularies and ontologies, such as the Environment Ontology^17^ and Disease Ontology^18^, as specified in the MIxS standard. Each term in the MIxS standard is defined to clarify the scope of each data descriptor.

When researchers neglect to submit enriched contextual metadata, is it because they do not realize the broader impact of their actions or they are unable to assess the benefits of describing their samples and study in comparison to the costs? Or is it that the benefits accrue as a social good and individual researchers receive little recognition and therefore tend to invest their valuable time elsewhere? One hopes the reason is not because they are withholding information over concerns of their data being reused as they are finalizing their own publications. Whatever the reasons, one consequence of ‘market failure’ in the supply of quality [omic] data is our inability to confidently compare and combine datasets, as the biological signals can be obscured by dominating, yet unaccounted, experimental confounding factors due to the absence of accurate and comprehensive metadata. For example, the effectiveness of state-of-the-art computational approaches – such as machine learning – are limited if the key signals (both biological and artifactual) in training datasets cannot be appropriately modelled. Yet, increasing statistical power through the analysis of large datasets or the application of machine learning approaches could help guide solutions to many of society’s greatest challenges.

As we solve these problems (technological and sociological) to achieve more complete metadata, it may be possible to identify datasets that are likely to hold previously un-investigated coronavirus sequence data and therefore possible insights into the natural reservoir of this currently important group of viruses. With more complete metadata it may be possible to ascertain the taxonomic, sequence, and environmental breadth of environmental viral genomes, thus providing insight towards future viral outbreaks. Community-driven consensus of data types and genomic standards informs infrastructure development and addresses the critical need for metadata standardization to mitigate duplication of effort and to enhance data sharing across outbreak investigations.

**When** the next global outbreak crisis occurs, we need a predefined, widely adopted multidimensional approach to organize critical genomic data. Our strategy to broadly inform how to clearly describe genomic metadata and the tools to prepare genomic metadata datasets needs to be expanded now. Our community needs the organizational ability and coordination to respond to the imminent need well in advance. Opportunities for coordination of reported data types are critical for data interoperability as contact tracing efforts and outbreak resources, such as Nextstrain^19^ and GISAID^20^ are being developed.

To move forward as a research community, we must restructure how we recognize and reward these efforts of broad societal value. We must call on researchers to “***provide a clear description of your data”*** and incentivize good data management plans that include the standardized collection of genomic metadata. We must also ensure that institutes and organizations adopt policies encouraging good metadata practices. Standards are consensual social technologies that necessarily take time to develop and require appropriate levels of reward (such as measures of data impact through reuse) when they are conformed to, but the current models for measuring output in academia (i.e. the number of peer-review citations) tend to overlook data contributions. Innovation begets new and improved standards supporting resilience of complex knowledge-driven societies. Decisive action is critical for development of essential genomics infrastructure. If we do not take decisive action, we will not be prepared.

In the words of Benjamin Franklin: “By failing to prepare, you are preparing to fail.”
